# Cultured epidermal autografts for treatment of stable vitiligo: Quantitative analysis of color matching with surrounding normally pigmented skin

**DOI:** 10.1111/1346-8138.16028

**Published:** 2021-06-25

**Authors:** Kazuhiro Toriyama, Hiroshi Kato, Hideyoshi Sato, Tomoyo Tanaka, Masukazu Inoie, Akimichi Morita

**Affiliations:** ^1^ Department of Plastic & Reconstructive Surgery Nagoya City University Graduate School of Medical Sciences Nagoya Japan; ^2^ Department of Geriatric and Environmental Dermatology Nagoya City University Graduate School of Medical Sciences Nagoya Japan; ^3^ Japan Tissue Engineering Co. Ltd Gamagori Japan

**Keywords:** color difference, color matching, cultured epidermal autograft, repigmentation, vitiligo

## Abstract

Cultured epidermal autografts (CEA) are surgical therapeutic alternatives for patients with stable vitiligo resistant to conventional medical treatments. In the present study, we assessed color matching before and at 12 months after CEA treatment. Eleven patients with 16 vitiligo lesions were included in this prospective study. The recipient sites were prepared by CO_2_ laser superficial ablation and subjected to CEA application. We clinically evaluated and categorized the color matching of the repigmented skin as well as the percentage of repigmentation. We also obtained three color values (L*a*b*) for the vitiligo lesions and surrounding normally pigmented skin. We then calculated the color differences between the two regions and compared them before and at 12 months after treatment. The mean percentage of repigmentation was 63.3% at 12 months. Six of the 16 lesions were categorized as “same as” and had color difference values of ≤5 at 12 months after treatment. Clinical evaluation of the color matching coincided well with the calculated color difference values. CEA application after CO_2_ laser superficial ablation was useful for treating vitiligo assessed by the percentage of repigmentation and color matching. Quantification of color differences may be a useful parameter for evaluating color matching in vitiligo.

**Abbreviations**
CEA: cultured epidermal autograft
CO_2_
: carbon dioxide

## 1 INTRODUCTION

Color difference was one of the parameters for evaluating color matching in vitiligo.^1^ The aim of the present study was to investigate whether cultured epidermal autograft (CEA) application after CO_2_ laser superficial ablation is useful for treating vitiligo by clinically and quantitatively assessing the repigmentation with color differences.

## 2 METHODS

### 2.1 Design of the intervention

The study was approved by the Ethics Committee of Nagoya City University Hospital (#46‐16‐0014). All patients provided written informed consent.

Eleven patients with 16 vitiligo lesions^2^ that were stable for at least 6 months and unresponsive to conventional therapies were enrolled in this prospective study (Table 1).

**TABLE 1:** Characteristics of the patients

Case	Age (years)	Sex	Type	Treated area	Previous treatments	Graft area (cm^2^)
1	48	M	NSV	Elbow	PT	5.5
2	58	F	NSV	Chest	EL, maxacalcitol	35.9
				Hand		51.6
3	30	F	NSV	Neck	PT, calcipotriol, maxacalcitol, MG	55.1
4	16	F	S	Abdomen	NB‐UVB, MG	106
5	29	F	S	Glabella	EL, MG	5.2
				Cheek		5
6	22	M	S	Mentum	NB‐UVB, MG	11.8
7	48	F	F	Forehead	NB‐UVB	5.7
8	40	M	S	Hands	SO, EL	28.1
9	53	M	S	Abdomen	PT	81.1
10	13	F	NSV	Lip	Tacrolimus, maxacalcitol, EL, MG	1.3
				Neck		1.7
				Clavicula		2.9
11	16	M	NSV	Lip	Maxacalcitol, MG	3.3
				Neck		4

Note.

Sex: M, male; F, female. Type: NSV, non‐segmental form; S, segmental; F, focal. Previous treatments: PT, phototherapy; EL, excimer laser; MG, mini‐grafting; NB‐UVB, narrowband ultraviolet B; SO, steroid ointment.

CEA were produced by Green’s culture technique at Japan Tissue Engineering.^3^ The epidermis of the affected area was removed by superficial ablation with a CO_2_ laser AcuPluse™ (Lumenis). Cultured autografts were then applied and occluded with silicon products. The treated area appeared red, and steroid ointment was applied topically. Patients were asked to expose the area to sunlight at home for 5 min daily with gradual progression to a maximum of 30 min daily after 3 months postoperatively.^3^ The patients were followed up at 1 week, and at 1, 3, 6, and 12 months.

Percentage of repigmentation was determined by planimetry using the VECTRA H1^®^ (Canfield Scientific) at 12 months after treatment.^4^ The repigmented skin was clinically evaluated and the color was categorized as “somewhat lighter than”, “same as”, or “somewhat darker than” the surrounding skin.^1^, ^5^ Three color values (L*a*b*) were obtained for the vitiligo lesions and surrounding normally pigmented skin using a digital camera (EOS Kiss X6i; Canon) after calibrating the images with a color reference marker, Casmatch™ (http://www.Bearmedic‐en.com), and Adobe Photoshop 2021 (Adobe Systems).^6^, ^7^, ^8^ We selected three directions centering on vitiligo, such as the cranial, the temporal, and the caudal, and measured the color values once in the peripheral part in each direction. We also measured the values once in the vitiligo region in the same direction. The respective color values were then averaged, and the color difference (ΔE*ab) between the lesions and the surrounding skin was calculated according to the formula.^6^ We then compared them before and at 12 months after treatment at the same anatomical points.

### 2.2 Statistical analysis

The influence of age, sex, and lesion location on the percentage of repigmentation was evaluated by the Mann–Whitney *U*‐test and Kruskal–Wallis test. Improvement in the color difference was evaluated by the Wilcoxon signed‐rank test. Percentage of repigmentation and ΔE*ab at 12 months and clinical evaluation of color matching were evaluated by Pearson’s correlation coefficient or point‐biserial correlation coefficient. The influence of age, sex, and lesion location on the improvement in color difference was evaluated by the Fisher exact test. *p* < 0.05 was considered statistically significant.

## 3 RESULTS

The results are summarized in Table 2. The clinical evaluations of the color matching coincided well with the calculated color difference values (*r* = −0.65). Lesion location affected the extent of the improvement in the color difference (*p* = 0.0253), but not age or sex. Locations such as the cheek, forehead, glabella, and lip tended to show more marked improvement in the color difference.

**TABLE 2:** Results

Percentage of repigmentation^*^	Clinical evaluation of color matching^†^	ΔE*ab	
		Before	At 12 months^‡^
12	Lighter	29.5	18.3
19	Lighter	12.9	11.4
1	Lighter	11.5	9.2
84	Lighter	13.5	3.6
100	Same	24.9	5.0
100	Same	10.7	4.9
100	Same	9.8	3.3
99	Same	9.2	2.1
75	Lighter	9.0	7.4
14	Lighter	9.4	7.8
80	Lighter	12.4	9.0
92	Lighter	8.7	5.5
68	Same	9.8	4.8
99	Same	6.8	3.3
23	Lighter	16.9	9.0
47	Lighter	16.4	11.1

Note.

Clinical evaluation:“lighter”, “somewhat lighter than”; same, “same as”.

Pearson’s correlation coefficient between * and ^‡^, −0.77.

Point‐biserial correlation coefficient between * and ^†^, 0.66.

Point‐biserial correlation coefficient between ^†^ and ^‡^, −0.65.

**FIGURE 1:**
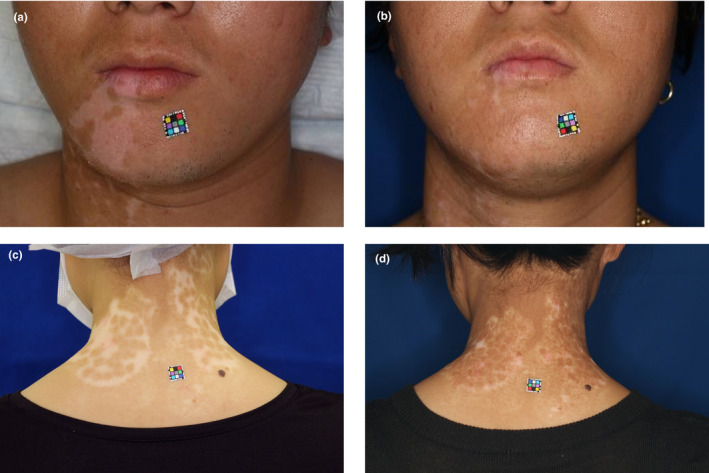
Case presentation. Case 6, mentum, 22‐year‐old male. (a) Preoperative. ΔE*ab = 9.2. (b) Twelve months after treatment. Repigmentation is 99%, clinical category is “same as”, and ΔE*ab = 2.1. Case 3, neck, 30‐year‐old female. (c) Preoperative. ΔE*ab = 13.5. (d) Twelve months after treatment. Repigmentation is 84%, clinical category is “somewhat lighter than”, and ΔE*ab = 3.6

There was two‐case presentation who had a uniform pigmentation and a patchy pattern (Figure 1). Areas of patchy pigmentation had a lower color difference number after averaging compared with the clinical evaluation.

## 4 DISCUSSION

We quantitatively assessed color differences of the treated skin with the surrounding skin to evaluate color matching. A spectrophotometer,^9^, ^10^ colorimeter,^9^ or digital camera^7^, ^8^ can be used to objectively assess color and color differences. In the digital images obtained by a camera, there is non‐uniformity related to the local conditions, such as the distance from object to illumination.^7^ Therefore, we used a photographic studio with the camera body and ring flash set at a fixed distance from the subject. We also adjusted the images using Casmatch™ and Photoshop™.^8^ We averaged each of the three color values because the lesion color was often uneven. Finally, and most importantly, we did not evaluate the color values themselves, but rather evaluated the differences in color between the lesions and the surrounding skin because using the differences in color reduced the impact of any remaining non‐uniformity related to the local conditions.

The present study has some limitations of the small sample size and non‐standardized analysis.

## CONFLICT OF INTEREST

T.T. and M.I. are employees of Japan Tissue Engineering. K.T., H.K., H.S., and A.M. have no conflicts of interest.
